# In Humans, Insulo-striate Structural Connectivity is Largely Biased Toward Either Striosome-like or Matrix-like Striatal Compartments

**DOI:** 10.1177/26331055241268079

**Published:** 2024-09-10

**Authors:** Adrian T Funk, Asim AO Hassan, Jeff L Waugh

**Affiliations:** 1 Division of Pediatric Neurology, Department of Pediatrics, University of Texas Southwestern, Dallas, TX, USA; 2 Department of Natural Sciences and Mathematics, University of Texas at Dallas, TX, USA; 3 Martinos Center for Biomedical Imaging, Massachusetts General Hospital, Charlestown, MA, USA

**Keywords:** Insula, striatum, striosome, matrix, probabilistic diffusion tractography, classification targets tractography

## Abstract

The insula is an integral component of sensory, motor, limbic, and executive functions, and insular dysfunction is associated with numerous human neuropsychiatric disorders. Insular efferents project widely, but insulo-striate projections are especially numerous. The targets of these insulo-striate projections are organized into tissue compartments, the striosome and matrix. These striatal compartments have distinct embryologic origins, afferent and efferent connectivity, dopamine pharmacology, and susceptibility to injury. Striosome and matrix appear to occupy separate sets of cortico-striato-thalamo-cortical loops, so a bias in insulo-striate projections toward one compartment may also embed an insular subregion in distinct regulatory and functional networks. Compartment-specific mapping of insulo-striate structural connectivity is sparse; the insular subregions are largely unmapped for compartment-specific projections. In 100 healthy adults, diffusion tractography was utilized to map and quantify structural connectivity between 19 structurally-defined insular subregions and each striatal compartment. Insulo-striate streamlines that reached striosome-like and matrix-like voxels were concentrated in distinct insular zones (striosome: rostro- and caudoventral; matrix: caudodorsal) and followed different paths to reach the striatum. Though tractography was generated independently in each hemisphere, the spatial distribution and relative bias of striosome-like and matrix-like streamlines were highly similar in the left and right insula. 16 insular subregions were significantly biased toward 1 compartment: 7 toward striosome-like voxels and 9 toward matrix-like voxels. Striosome-favoring bundles had significantly higher streamline density, especially from rostroventral insular subregions. The biases in insulo-striate structural connectivity that were identified mirrored the compartment-specific biases identified in prior studies that utilized injected tract tracers, cytoarchitecture, or functional MRI. Segregating insulo-striate structural connectivity through either striosome or matrix may be an anatomic substrate for functional specialization among the insular subregions.

## Introduction

The human insula is a heterogeneous cortical region that is surrounded by the frontoparietal operculum, the temporal operculum, and the Sylvian fissure.^1^ The known functions of the insula are diverse, including interoception, visceral motor, vestibular, heat and pain recognition,^2^ emotional and cardiovascular regulation, and auditory and language processing.^3^ This range of functions is in part mediated by subregional specialization within the insula. The insula can be divided into distinct subregions based on gyral architecture, cytoarchitectural features (neuronal density and subtype), or functional connectivity.^4-8^ For example, cytoarchitectural characterization in multiple mammalian species identified 3 insular regions distinguished by patterns of neuronal density: the granular (caudodorsal), agranular (rostroventral), and dysgranular (intermediate) subdivisions.^5^ Similarly, segmenting the insula based on covariance in resting state functional connectivity yields 3 subdivisions: dorsal anterior, with connectivity to frontal, anterior cingulate, and parietal cortices; ventral anterior, with connectivity to limbic and affective areas; and mid-posterior, with connectivity to sensorimotor and nociceptive areas.^6-9^ Insular subregions defined by functional connectivity closely align with segmentations based on cytoarchitectural gradients, underscoring the links between insular structure and function.

The insula displays extensive cortical and subcortical structural connectivity. Insulo-striate connectivity is particularly extensive, is somatotopically organized, and correlates with distinct insular functions, such as reward anticipation and adolescent decision-making.^10-14^ Insulo-striate projections terminate in 2 structurally and functionally distinct tissue compartments: the striosome and matrix. While both compartments are comprised of medium spiny projection neurons (MSNs), these populations migrate to the striatum at different developmental timepoints^15,16^ via different classes of radial glia,^17^ they have segregated vascular supplies,^18^ they differentially express more than 60 histochemical markers,^19^ and have different afferent and efferent patterns of structural connectivity, as revealed by injected anterograde and retrograde tracers (reviewed in Waugh et al^14^).

Striosome and matrix appear to be functionally distinct. They are differentially activated during value-driven learning, affective judgments, and action selection.^20-22^ In self-stimulation paradigms, rats acquire and maintain lever-pressing responses significantly more when electrodes contact striosomes than when they contact matrix.^23^ The compartments also have divergent responses to dopamine release^24^ and differential activation following exposure to psychomotor stimulants, such as cocaine and methamphetamine.^19,25-29^ Although the precise location of striosome and matrix varies between individuals, the striosome is generally enriched in the rostro-ventro-medial striatum, while the matrix is concentrated in the caudo-dorso-lateral striatum.^26-31^ Anterograde tracer injections in the rostral and ventral insular cortex of cat and macaque resulted in selective innervation of the striosome, with sparse innervation of the matrix.^28,32^ A recent publication demonstrated that the human insula follows this same pattern of region-specific compartment bias,^14^ though comprehensive and quantitative mapping of compartment-specific connectivity across all insular subregions was beyond the scope of that publication.

Although the somatotopy of insulo-striate structural connectivity has been studied previously, compartment-specific insular connectivity has not been mapped or quantified, to the best of our knowledge. Given the diversity of functions attributed to the insula, the substantial cytoarchitectural differences between its subregions, and the potential for compartment-specific insulo-striate projections to link neighboring insular subregions with distinct cortico-striato-thalamo-cortical (CSTC) loops,^33,34^ the objective of this paper was to quantitatively map the structural connectivity of the insular subregions with each striatal compartment. In 100 healthy subjects from the Human Connectome Project (HCP) the previously established diffusion tractography method^14^ was utilized to segment the striatum into voxels with striosome-like and matrix-like patterns of structural connectivity. Biases in structural connectivity were then quantified between 19 anatomically segmented insular subregions^35^ and the striosome-like and matrix-like compartments. These investigations revealed that insulo-striate streamlines that contact striosome-like versus matrix-like voxels were spatially segregated. The insular zones that displayed compartment-specific bias overlapped with the insular subdivisions previously identified through histologic and MRI-based assessments. This segregation of insulo-striate structural connectivity into compartment-specific networks may contribute to the diverse functions of the human insula, and to the specific symptoms associated with neurodegenerative diseases that affect the insula and/or striatum. Mapping compartment-specific biases in structural connectivity is an essential step in understanding the role of insulo-striate projections in human health and disease.

## Materials and Methods

### Overview

In a cohort of 100 healthy subjects, a previously-established probabilistic diffusion tractography method was utilized to identify striatal voxels with striosome-like and matrix-like patterns of structural connectivity.^14^ Following striatal parcellation, 8 subsequent rounds of probabilistic tractography were used to validate the striatal parcellations, map compartment-specific insulo-striate streamlines, quantify bias in insulo-striate connectivity, and assess alternate anatomic contributions to the biases in structural connectivity that were described. All research was conducted in accordance with the principles set forth in the Declaration of Helsinki and was approved by our University’s Institutional Review Board.

### Experimental cohort

An experimental cohort of 100 healthy subjects was assembled, totaling 200 hemispheres, from the Human Connectome Project dataset,^36-38^ accessed through the National Institute of Mental Health Data Archive (NDA).^38^ This cohort was racially diverse, included broad representation across the adult age span, and was balanced for sex. Ten HCP subjects were identified (5 female, 5 male) at 5-year intervals, beginning at 20 years old and ending at 65 years old (20, 25, 30, etc.). If a particular age block had an insufficient number of HCP subjects to reach our 10-subject goal, participants in adjacent ages were utilized, but never crossed into an adjacent 5-year interval. Within each 10-subject block (5 female, 5 male), a diverse racial representation was created by including at least 1 Asian and at least 1 Black individual (as defined by the subject) for each sex at each age block. For any age-sex block in which an insufficient number of subjects who identified as a particular race was available, either the adjacent age block or the opposite sex from the same age block was used to fill these groups. The remainder of each age/sex block with was filled with White subjects, which were abundant in the HCP database. In 6 of 10 age blocks, there were insufficient subjects available to meet all of the target criteria (a diverse group of 5 females, 5 males), so the opposite sex for that age block was overfilled to reach the 10-subject goal and such mismatches were balanced in adjacent age blocks. This 100-subject cohort was therefore evenly split between females and males (50:50). Subjects self-reported their race as: 13% Asian, 28% Black, 51% White, and 8% Multiracial/Other/Not reported. The average age of these subjects was 42.3 years (age range: 20-65 years). Based on the Edinburgh Handedness Inventory,^39^ 90 subjects were right-handed (90%) and 10 were left-handed (10%). All 100 subjects were healthy, with no recognized neurologic or psychiatric conditions. A pre-set criteria was imposed for including a hemisphere in our final analyses (see 2.4: Insular Segmentation, below), which eliminated 4 hemispheres. No subject had both hemispheres eliminated.

### Background—striatal parcellation method

In 2022, we described a method to parcellate the striatum into striosome-like and matrix-like compartments in vivo in humans.^14^ This method was further developed to assess compartment-specific striato-pallido-thalamic projections, demonstrating that most thalamic nuclei have compartment-specific biases in structural connectivity.^40^ In brief, striatal parcellation leverages the anatomic findings from 4 decades of histologic investigation in animals, which used injected tract tracers to map compartment-selective projections in mice, rats, cats, dogs, and non-human primates. Extra-striate regions with compartment-selective afferents in animals were selected and organized into groups that favored either the striosome or matrix compartments. These groups were used as “bait” regions for quantitative probabilistic tractography. For each striatal seed voxel, the abundance of streamlines that reached striosome-favoring versus matrix-favoring bait regions defined that voxel’s compartment-specific connectivity profile. The most-biased voxels (those whose connectivity bias was ⩾1.5 standard deviations above the mean for the striosome-like and matrix-like probability distributions) were selected to represent the compartments in subsequent quantitative assessments of insulo-striate structural connectivity. Given the inferential nature of this method, the parcellated striatal voxels will henceforth be referred to as “striosome-like” or “matrix-like.”

The reliability of connectivity-based parcellations was high; test-retest comparisons in scans performed 1 month apart identified an error rate of 0.14%.^14^ Voxels identified as striosome-like or matrix-like were unlikely to convert between compartments in a subsequent scan. These voxels were defined based solely on their connectivity profiles, but then assessed other anatomic features of the striosome and matrix (as reported from the animal and human histology studies cited above) to check the validity of these connectivity-based striatal parcellations: their spatial distribution, relative abundance, and connectivity with regions that were mapped in animals but were left out during striatal parcellation. In every case, our striosome-like and matrix-like voxels shared the anatomic features demonstrated through injected tract tracers and histochemistry. However, readers should bear in mind that this method is probabilistic and indirect—striosome and matrix tissue was not directly identified using this method.

### MRI acquisition and processing

This study was a secondary analysis of existing HCP MRI data. All subjects were scanned at 3 Tesla with a whole-brain diffusion tensor imaging (DTI) protocol. All MRI data was collected in a single scan session. All subjects were scanned using Siemens Prisma scanners running Syngo MR E11 software and using harmonized protocols at 3 separate sites in the United States.^37^ DTI for these subjects was obtained at 1.5 mm isotropic resolution using 200 diffusion directions (14 B_0_ volumes, 186 volumes at non-colinear directions) with the following parameters: repetition time = 3.23 seconds; echo time = 0.0892; imaging volume = 256 mm × 256 mm × 256 mm. Paired anterior-posterior and posterior-anterior DTI volumes were used to estimate and correct for susceptibility-induced field distortions using the FSL tool *topup*. Skull stripping and motion correction was performed using the FSL Brain Extraction Tool (*bet2*). The FSL tool *eddy_openmp* was used to correct for eddy current distortions and head motion. The FSL tool *bedpostx* was then used to fit probability distributions on diffusion parameters at each voxel, including modeling of crossing fibers. Using *dtifit*, local diffusion tensors were fit to create 3D FA images at 1.5 mm isotropic resolution. All DTI preprocessing and tractography steps were completed in each subject’s native diffusion space, utilizing the University’s distributed Linux cluster.

### Insular segmentations

The insular segmentations of Ghaziri et al^35^ were utilized to extract connectivity measures from probabilistic tractography. This atlas was selected because the ROI segmentation offered substantial granularity, its subregions conformed to human gyral anatomy, and it was derived using the same (diffusion) methodologies utilized in the present study. This segmentation technique parcellated the human insular cortex into 19 distinct subregions for each hemisphere using a K-means random parcellation algorithm followed by manual segmentation of the insula to harmonize subregions with sulco-gyral divisions. These segmentations were graciously shared by the D. Nguyen lab, University of Montreal, Canada. These subregional segmentations were used in MNI standard space (1 mm isotropic voxels) to extract connectivity measures. Registration of these insular subregion masks into native diffusion space were determined to result in boundary erosion between the adjacent masks, leading to occasional gaps or overlap. If the insulo-striate connectivity were measured in native space, this loss of precision would yield incomplete and inaccurate sampling. Therefore, the tractography-derived probability maps (that were generated in native diffusion space) were registered into MNI standard space for quantification of tractography for each insular subregions.

### Anatomic masks

Seed, waypoint, and bounding masks were defined in MNI standard space and subsequently registered these masks into subjects’ native diffusion space. All tractography was completed in native diffusion space. The caudate and putamen were manually segmented, excluding the nucleus accumbens. The caudal half of the tail of the caudate was also excluded, as registration errors and partial volume effects reduced the accuracy of registration for this narrow tail.^14^ A whole-insula seed for tractography was generated by summing all insular subregions,^35^ inflating that volume by 1 voxel in all dimensions, and then manually trimming the mask relative to the MNI152_T1_1mm standard. An insula-subcortical bounding mask was generated to refine the tractography, which encompassed the insula, caudate, putamen, and the white matter surrounding these structures. Pilot rounds of tractography were used to refine this bounding mask, with visual inspection of each subject and hemisphere to ensure that this mask did not exclude valid insulo-striate streamlines. This mask eliminated all streamlines that extended beyond its boundaries, excluding non-insular corticostriate, striatopallidal, thalamostriate, and striatal-brainstem projections. All other regions of interest (ROIs), including bait regions, striatal segmentations, and our midline exclusion mask, were identical to those detailed in our original description of this method.^14^ The bait, seed, waypoint, and bounding masks can be accessed here: github.com/jeff-waugh/Striatal-Connectivity-based-Parcellation.

### Probabilistic tractography

Seven rounds of probabilistic tractography were completed in this study (Table 1). The goals of each round will be summarized briefly, with a deeper background on each round of tractography provided in subsequent paragraphs. First, the striatum was parcellated into striosome-like and matrix-like voxels, based on differential structural connectivity (tractography round 1, see Methods 2.3). The anatomic features (detailed below) of these parcellated striatal voxels were measured to assure that our striosome-like and matrix-like masks shared the anatomic features of striosome and matrix observed in tissue. Tractography rounds 2 and 3 were part of this validation step. Compartment- level biases in cortico-striate projections were assessed matched the biases measured through injected tract tracers in animals: parcellating with 1 bait region left out (an N-1 parcellation, tractography round 2), followed by quantification of compartment-specific bias in the left-out region (round 3). These voxels served as the seeds or targets for rounds of tractography that tested our hypotheses (rounds 4-7). Round 4 mapped the location of insulo-striate streamlines, comparing streamlines that targeted striosome-like vs. matrix-like targets. Round 5 quantified compartment-specific biases in insulo-striate structural connectivity for each of the 19 insular subregions. Rounds 6 to 7 were post-hoc tests of alternative hypotheses, assessing non-compartment drivers of connectivity bias. Round 6 considered the possibility that nucleus-of-origin (caudate vs. putamen) might influence compartment-specific biases in insulo-striate connectivity. Round 7 tested the hypothesis that local anatomic features of the striatum, separate from compartment-level bias, might explain our insulo-striate findings.

**Table 1.** Summary of the 7 rounds of tractography utilized to test our experimental aims. Rounds were organized sequentially, based on the experimental goals that served those aims. “Type of Tractography” refers to either traditional streamline tractography or classification targets tractography. Rounds 1 to 3 (green) established and validated our striatal compartment parcellations, rounds 4 and 5 tested our primary hypotheses regarding insulo-striate connectivity (white), and rounds 6 and 7 tested potential alternate explanations for our findings (blue).

Round	Type of tractography	Seed region	Target region	Goal of tractography
1	CTT	Striatum	Extra-striate Bait Regions	Parcellate striatum into striosome-like, matrix-like voxels
2	CTT	Striatum	Extra-striate N-1 Bait Regions	Test accuracy of striatal parcellations from Round 1
3	CTT	N-1 Region	Compartment-specific N-1 Striatal Masks	Test accuracy of striatal parcellations from Round 1
4	Streamline	Whole Insula	Compartment-specific Striatal Masks	Map compartment-specific insulo-striate streamlines
5	CTT	Whole Insula	Compartment-specific Striatal Masks	Quantify biases in insulo-striate connectivity
6	CTT	Whole Insula	Caud/Put-Specific Compartment Masks	Assess compartment-specific vs. nucleus-of-origin effects
7	CTT	Whole Insula	Compartment-adjacent Striatal Masks	Assess compartment-specific vs. neighborhood effects

Abbreviations: Caud, caudate; CTT, classification targets tractography; Put, putamen.

All rounds of tractography were executed in each subject’s native diffusion space. The following probtrack × 2 settings were used for all tractography: curvature threshold = 0.2; steplength = 0.5 mm; number of steps per sample = 2000; distance correction, to prevent target proximity from influencing connection strength. Rounds 1 and 2 (striatal parcellation) used a whole-hemisphere mask to bound the tractography; rounds 3 through 9 (insulo-striate tractography) used the previously described insulo-striate bounding mask that restricted streamlines to only those transiting the insula, striatum, and surrounding white matter, eliminating streamlines that strayed outside the insulo-striate path. Rounds 1, 2, and 4 produced adequate samples by seeding with 5000 streamlines per seed voxel, while rounds 3, and 5 to 7, required an increase to 50 000 streamlines per seed voxel to adequately quantify connectivity within their seed volumes.

In tractography round 1, classification targets tractography (CTT) was utilized to parcellate the striatum into striosome-like and matrix-like voxels (see Methods “Background – striatal parcellation method”). Briefly, sets of striosome-favoring and matrix-favoring regions were used as “bait” for CTT. This set of bait regions, which included the rostral insula, produced robust striatal parcellations.^14^ However, one cannot use a region to parcellate the striatum and then accurately quantify connectivity with that region. The insula was therefore removed from the set of bait regions and performed striatal parcellation with the remaining 4 striosome-favoring regions (mediodorsal thalamus, posterior orbitofrontal, basolateral amygdala, and basal operculum) and 5 matrix-favoring regions (supplementary motor area, primary motor cortex, primary sensory, globus pallidus interna, and the VLc/VPLo thalamic nuclei). This parcellation method is identical to one that we utilized previously,^14^ identified there as an “N-1 analysis.”

Following striatal parcellation, voxels from the tails of the probability distribution were selected to generate equal volume masks of the most-biased striosome-like and matrix-like voxels. To eliminate the possibility that differing target size could skew streamline quantification, the striosome-like and matrix-like masks in each hemisphere, and in each individual, were always of equal volume. A fixed volume standard was set for inclusion (the uppermost 13% of voxels from each distribution, equivalent to 1.5 standard deviations above the mean in a normal distribution) from each subject’s striosome-like and matrix-like probability distributions. These striosome-like and matrix-like masks were utilized, in subsequent steps, as the seeds or targets for other rounds of tractography.

Before proceeding to insulo-striate mapping, the features of our striatal parcellations were compared to the expected relative abundance and location of striosome and matrix, established previously through histology. The volumes of striosome-like and matrix-like voxels that reached substantial bias were measured (connection probability ⩾ 0.87, to parallel the 1.5 standard deviation threshold noted above). For each hemisphere and subject, these measures were expressed as the percentage of all supra-threshold volume (compartment X/(compartment X + compartment Y)). Since the distributions of striosome and matrix differ in their intrastriate location (as demonstrated through histology), the locations of these compartment-like voxels were measured as another way to assess the accuracy of our parcellations. For each voxel in our striosome-like and matrix-like masks, its cartesian position was measured relative to the centroid of the nucleus it resided in (caudate or putamen) for each subject and hemisphere, producing a dataset of 66,812 parcellated voxels. Finally, compartment-specific bias in structural connectivity was quantified for primary motor cortex and posterior orbitofrontal cortex, regions with robust matrix-favoring and striosome-favoring bias, respectively.^27,28,41^ Just as N-1 parcellations were performed to leave out the rostral insula (tractography round one), for tractography round 2 striatal parcellation was performed with either the primary motor or posterior orbitofrontal cortices left out. The most-biased voxels for each of these N-1 parcellations were then identified (equal volume striosome-like and matrix-like masks, distinct from those generated for tractography round one) to serve as targets for quantitative CTT (tractography round 3).

Tractography round 4 mapped insulo-striate streamlines with 2 iterations, alternating the seed and target ROIs (A-to-B and B-to-A) and averaging the results (AB-BA). First, the whole insula was used as seed and either striosome-like or matrix-like voxels (in separate iterations) as an obligatory waypoint. Second, striosome-like or matrix-like voxels were set as seeds (in separate iterations) and the whole insula as an obligatory waypoint. This round of tractography was performed in both directions (whole insula to compartment-specific voxels, and compartment-specific voxels to whole insula) to reduce potential bias from the directions of diffusion acquisitions and to increase the reliability of streamline mapping. The output of these 2 iterations (A-to-B–B-to-A) were then averaged for each subject and hemisphere. The output of this round of tractography (streamline distributions that were largely extra-insular) allowed for the quantification and mapping of streamline bundles that connected the insula to each striatal compartment. The striosome-like and matrix-like versions of these runs of tractography utilized the same insular seed mask, seeded the same number of streamlines per insular voxel, were bounded by the same insulo-striate bounding mask, utilized equal volume striatal masks, and corrected for any path length differences between seed and target voxels.

In tractography round 5, compartment-specific connectivity was mapped within the insula by performing CTT with the insula as seed and the 2 striatal compartments as targets. The output of this round of tractography, striosome-favoring and matrix-favoring probability maps of the insula, allowed for the quantification of connectivity at each insular voxel, and thus for each insular subregion. Utilizing the standard *probtrackx2* settings for streamline seeding (5000 streamlines per seed voxel) left some subjects, and some subregions of the insula, relatively undersampled. Increasing the depth of sampling to 50 000 streamlines/voxel yielded a more robust probability distribution, and therefore, more accurate quantification of connectivity. Specifically, subsequent quantification steps showed that artificial binarization of the connectivity data (when suprathreshold voxels were all striosome-like, or all matrix-like) led to less accurate connectivity estimates. Increasing the number of streamlines per seed voxel reduced the number of subjects and insular subregions with zero connectivity for 1 striatal compartment, and thus reduced this artificial binarization.

After assessing our primary aims with these 5 rounds of tractography, it was evident that the measures of compartment-specific insular connectivity might have been influenced by relative differences in regional connectivity—that striosome vs. matrix differences might have been influenced by caudate versus putamen differences in structural connectivity. A post-hoc round of tractography (round 6) was carried out, aimed at understanding the influence of caudate/putamen connectivity on striosome/matrix connectivity. Round 6 differed from round 5 only in the location of the compartment-like target masks. For this iteration, the same striatal parcellations (probability maps) that were generated in tractography round 1 were used, but we defined new striosome-like and matrix-like masks. Rather than choosing the most-biased voxels (uppermost 1.5SD) from the whole striatum, these masks were defined proportionally, adjusting for the relative volume of caudate and putamen. Specifically, instead of choosing the 180 most-biased voxels from any part of the striatum, the 80 most-biased voxels were selected from the caudate and 100 most-biased voxels from the putamen. For every combination of subject, hemisphere, and region, the volume of striosome-like and matrix-like voxels was equal. These regionally proportionate compartment masks were utilized as targets for CTT, which allowed for the mapping of connectivity between each insular voxel and the striatal compartments without the potential for caudate-putamen bias.

Tractography round 7 distinguished compartment-specific effects from the influence of the striatal “neighborhood” in which those voxels reside. That is, the objective was to learn whether other voxels that were nearby, but that were not selected based on biased connectivity, would also drive the biases in insulo-striate connectivity seen in tractography round 5. Round 7 was identical to round 5—CTT with the whole insula as seed—with 1 exception: instead of targeting the precisely-selected striosome-like and matrix-like masks, this tractography targeted the near-neighbors of these precisely-selected voxels. For every voxel in the compartment-like masks, the position in each plane was shifted by ±0 to 3 voxels at random, ensuring that no voxels in the randomly shifted mask were reselected from either original 1.5SD mask. This randomized relocation ensured that the precise-to-neighboring voxel change would maintain the topographic organization of our compartment-specific 1.5SD masks. The position of every shifted voxel was measured for comparison with the original 1.5SD voxels to assure that our masks had not shifted, on average, from the original “neighborhood.” CTT with the whole insula as seed and these location-shifted neighboring striatal voxels as targets was then utilized for quantification, identical to the assessment in round 5.

### Assessing the amplitude and location of insulo-striate streamlines

These results were generated using 2 different modes of tractography (traditional streamline in round 4 vs. CTT in rounds 1-3 and 5-9) and therefore required 2 different approaches to quantifying their outputs. For tractography round 4, the total number of insula-seeded streamlines that reached striosome-like or matrix-like voxels were quantified. That count was then normalized by the volume of that individual’s insular seed mask to reduce the impact of inter-individual differences in insula size. This count—the number of streamlines per insular voxel—was compared between striosome-favoring and matrix-favoring tractography for left and right hemispheres. The relative abundance of striosome-favoring versus matrix-favoring streamlines was biased toward the same compartment in both hemispheres, so the hemispheres were combined for subsequent quantification steps. Since the goal was to investigate biases in connectivity, rather than its absolute strength, connectivity was expressed as the percentage of streamlines that reached matrix-like voxels (matrix count/(striosome count + matrix count)). This formulation reduced the outsize influence of subjects whose total streamline counts were unusually high or low.

After registering each subject’s tractography into MNI space, averaged streamline tractography volumes were generated for each hemisphere and compartment. Distinguishing between the tract core and the penumbra of less-specific streamlines is critical when assessing the streamlines of probabilistic tractography.^42-45^ Each averaged tract was therefore normalized relative to its maximum amplitude with thresholds set to retain the uppermost 25%, 50%, or 75% of all voxels. The volume of each thresholded average was then assessed, and their volume of overlap, to determine the Dice similarity coefficient (DSC), as follows:

$$\frac{2\times ({Volume}_{Projection\;from\;Striosome\;Seed}\cap {Volume}_{Projection\;from\;Matrix\;Seed})}{{Volume}_{Projection\;from\;Sriosome\;Seed}+{Volume}_{Projection\;from\;Matrix\;Seed}}$$

### Assessing compartment-specific bias in insular subregions

For tractography iteration 5 (CTT, insula as seed, striosome-like, and matrix-like voxels as targets), the intra-insular locations of compartment-specific connectivity was assessed, as well as the mean connectivity bias for each of 19 insular subregions. First, the averaged insular probability distributions for striosome-favoring and matrix-favoring CTT was visually inspected. While matrix-favoring voxels occupied a single cluster, striosome-favoring voxels occupied 2, separated clusters in the rostral and caudal insula. The 2 clusters of striosome-favoring voxels were assessed independently, with each cluster compared to the position of the matrix-favoring cluster. For each individual and hemisphere, the center of gravity (COG, using *fslstats*) was identified within the matrix-favoring distribution, spanning the whole insula. For the striosome-favoring distribution, the insula was split at coronal plane *y* = 1 (MNI convention)—the center of the matrix cluster, and therefore, the nadir of the striosome-favoring distribution. The COGs within the rostral and caudal striosome-favoring distributions were then identified for each individual and hemisphere. The cartesian position of all COG measures was adjusted to account for differences in the position of the insula between left and right hemispheres, so that the left and right hemisphere data could be combined when measuring inter-compartment differences in COG location.

### Determining compartment specificity for insular subregions

Next, the degree of compartment specificity in each of 19 insular subregions was assessed. To improve the accuracy of our data extraction, a correction for partial volume effects induced by registration of probability maps into MNI space was completed. In native space, the striosome-like and matrix-like probability distributions sum to 1 at each voxel. After registration to MNI space, probability at some voxels no longer summed to one. Therefore, the striosome-favoring and matrix-favoring probability distributions needed to be normalized on a voxel-by-voxel basis. Edge voxels whose summed value (striosome plus matrix) was <0.5 was trimmed, which reduced partial volume effects at the edges of the insula. After normalization and edge trimming, the insular segmentations of Ghaziri et al^35^ were utilized to extract data for each of 19 insular subregions. Quantification measures were extracted for the number of suprathreshold voxels (value ⩾0.55) in striosome-favoring and matrix-favoring normalized probability distributions within each insular subregion. For each subregion, connectivity biases were expressed as the volume percent: the percent of suprathreshold voxels dominated by each compartment (N_voxels_, striosome or matrix/(N_voxels_, striosome + N_voxels_, matrix). For all insular subregions, the left and right hemispheres were biased toward the same compartment, so hemispheres were combined for this quantification step.

The method of quantifying compartment-specific bias could be compromised by undersampling of the probability distribution. For example, consider the addition of 1 marginal voxel in 2 subjects with the same compartment ratio—subject A, with 1 matrix-favoring voxel and 9 striosome-favoring voxels, and subject B, with 10 matrix-favoring voxels and 90 striosome-favoring voxels. Adding a single matrix-favoring voxel to each subject will increase the matrix bias by 8.2% for subject A, and 2.1% for subject B. To avoid non-linear influences on quantification, a minimum threshold of 19 suprathreshold voxels (the number of insular subregions considered) was established for each compartment-specific probability distribution. That is, to be included in our final cohort for quantification a hemisphere had to have at least 19 suprathreshold voxels for both striosome-favoring and matrix-favoring distributions. This minimum sample criteria led us to eliminate 4 hemispheres, for a final sample of 196 hemispheres. Note that the increase in the number of streamlines per seed voxel to 50 000 (a 10× increase over the standard settings) substantially decreased the number of undersampled hemispheres.

Tractography rounds 6 and 7 were post-hoc analyses to determine the influence of regional connectivity biases (caudate vs. putamen; precisely selected vs. neighborhood voxels) on compartment-specific connectivity biases. For these rounds, tractography was performed with the same parameters as round 5 (CTT with insular seed and striatal compartment targets), but with seed or targets adjusted to test alternate explanations for these insulo-striate findings. As in round 5, for rounds 6 and 7 compartment-specific connectivity was expressed as the volume percent projecting to either striosome-like or matrix-like voxels.

### Significance testing

Significance testing was performed using STATA (StataCorp, 2023, Stata Statistical Software: Release 18. College Station, TX). Two sets of comparisons were completed to assess the accuracy of our striatal parcellations: intrastriate voxel location, and compartment-specific bias in cortico-striate structural connectivity (N-1 analyses). Two-factor ANOVA was utilized to assess the effect of striatal compartment and nucleus of origin (caudate or putamen) on intra-striate voxel location (tractography round 1; separate ANOVAs for the *x*-, *y*-, and *z*-planes). Since subjects’ compartment-like mask volume differed from person to person depending on the availability of highly biased, compartment-specific voxels, subject identity was controlled for as a nuisance variable. We previously demonstrated that scanner type, subject sex, and self-identified race had no significant influence on voxel location.^14^ Therefore, this testing did not model these factors. Hemisphere was not included as a factor, as no interhemispheric differences in matrix and striosome location have been described previously, to the best of our knowledge. Analysis of simple main effects for factor interactions was completed using the SME utility (UCLA ATS Statistical Consulting Group).^46^ The conservative simultaneous test procedure, available through the SME utility, was used to estimate the F-critical. Compartment-specific biases in cortico-striate connectivity were assessed (tractography round 3) using 2 *t*-tests. Striosome-favoring and matrix-favoring voxels were compared (volume percent) for 2 regions, primary motor and posterior orbitofrontal cortices, and therefore utilized a significance threshold of *P* = .025 (Bonferroni correction for these and all subsequent comparisons; 0.05/2 tests).

Insulo-striate streamline counts were compared between the hemispheres (tractography round 4) using 3 *t*-tests (2 tailed, paired samples; left vs. right hemisphere, total streamlines; left vs. right hemisphere, matrix-favoring streamlines; left vs. right hemisphere, striosome-favoring streamlines). The significance threshold was therefore set at *P* = .0167 (0.05/3 tests). The location and amplitude of streamline bundles were compared (those that favored striosome-like vs. matrix-like voxels) using voxelwise, non-parametric testing (FSL’s *randomise*) with the following parameters: 5000 permutations; variance smoothing = 2 mm; threshold-free cluster enhancement mode; masked by the same subcortical bounding mask utilized to generate the streamlines (tractography round 4). Left and right hemisphere tractography volumes were combined into a single bilateral volume to reduce the number of *randomise* comparisons. As a subsequent round of *randomise* testing was completed with similar goals (described below), these were considered to be a family of tests and therefore utilized a significance threshold of *P* = .025 (0.05/2 tests). For all *randomise* comparisons, familywise error-correction and threshold-free cluster enhancement were utilized.

The intra-insular root-mean-square distance was compared between centers of gravity for striosome-favoring and matrix-favoring clusters (tractography round 5) using 1 sample *t*-tests with the assumption of no location difference between compartment-specific centers of gravity. Two of these one-sample tests were performed (anterior striosome-favoring vs. matrix-favoring; posterior striosome-favoring vs. matrix-favoring), so the significance threshold for root-mean-square comparisons was set at *P* = .025 (0.05/2 tests). The *x*-, *y*-, and *z*-plane locations were compared for the COGs of striosome-favoring and matrix-favoring clusters, testing anterior striosome-favoring vs. matrix-favoring, and posterior striosome-favoring vs. matrix-favoring COGs. Therefore, 6 *t*-tests were performed (2 tailed, paired samples), leading to a significance threshold of *P* = 8.3 × 10^−3^ (0.05/6 tests).

Each insular subregion was tested for compartment-level biases in structural connectivity through 2 related, but distinct approaches. Both approaches utilized the classification targets probability maps derived from tractography round 5 but tested these probabilities in different ways. First, a series of ANCOVAs were performed that tested for influence on the volume percent (the fraction of suprathreshold voxels that favored striosome-like or matrix-like voxels, described in section “Determining compartment specificity for insular subregions”). Collinearity among the insular subregion measures precluded the use of MANCOVA. The explanatory factors considered for each subregion included compartment, hemisphere, handedness, sex, self-identified race, and age. Given the recent findings by Cabeen et al^47^ that hemispheric lateralization in the rostral-most insula is significantly influenced by sex, the combined interaction of sex, hemisphere, and compartment was also included. As 19 separate ANCOVAs were performed (one for each insular subregion), the significance threshold was set at *P* = 2.6 × 10^−3^ (0.05/19). Next, the bias in structural connectivity at individual voxels was assessed across the whole insula with *randomise*, using the same parameters described for tractography round 4, but masking the analysis using the whole-insula region previously utilized as the seed for tractography. As this comparison was paralleled by the previously described iteration of *randomise*; both utilized a significance threshold of *P* = .025.

Finally, a series of post-hoc comparisons were performed to assess the influence of nucleus of origin or local striatal environment on compartment-specific biases (tractography rounds 6 and 7). Round 6 (testing the influence of nucleus of origin) utilized the same set of 19 ANCOVA utilized for tractography round 5, and therefore the significance threshold was set to equal our *a priori* ANCOVA comparisons (*P* = 2.6 × 10^−3^). All ANCOVA results that were <10 times the significance threshold (*P* = 2.6 × 10^−2^) were labeled as ‘trending’ toward significance. Insulo-striate compartment selectivity was assessed when projecting to location-shifted striatal masks (tractography round 7), using *t*-tests with a significance threshold of *P* = 2.6 × 10^−3^ (0.05/19).

## Results

### Study summary

This study investigated structural connectivity between the insular cortex and the striatum to determine whether particular insular subregions were biased in their connectivity with the striatal compartments, striosome and matrix. First, an analysis was performed to assess the degree to which our striosome-like and matrix-like voxels matched the anatomic properties of striosome and matrix observed in animal and human tissue. Next, we mapped the insular subregions for compartment-level bias in structural connectivity. The first step in these analyses was to assess the location and amplitude of insulo-striate streamline bundles after they exited the insula. Next, we evaluated each insular subregion for compartment-specific bias in structural connectivity. At the levels of the whole insula, the insular subregions, and the subcortical white matter, insulo-striate structural connectivity with striosome-like voxels had markedly different location and amplitude than connectivity with matrix-like voxels. For some subregions, compartment bias was significantly influenced by a sex-by-hemisphere interaction. Finally, insulo-striate connectivity was assessed for potential alternate anatomic factors, unrelated to the striatal compartments, that might also influence compartment-specific bias. Neither nucleus-of-origin (caudate or putamen) nor local striatal environment was a significant or plausible explanatory factor for the compartment-specific biases that were identified.

### The relative location of striosome-like and matrix-like voxels

**Figure 1.**
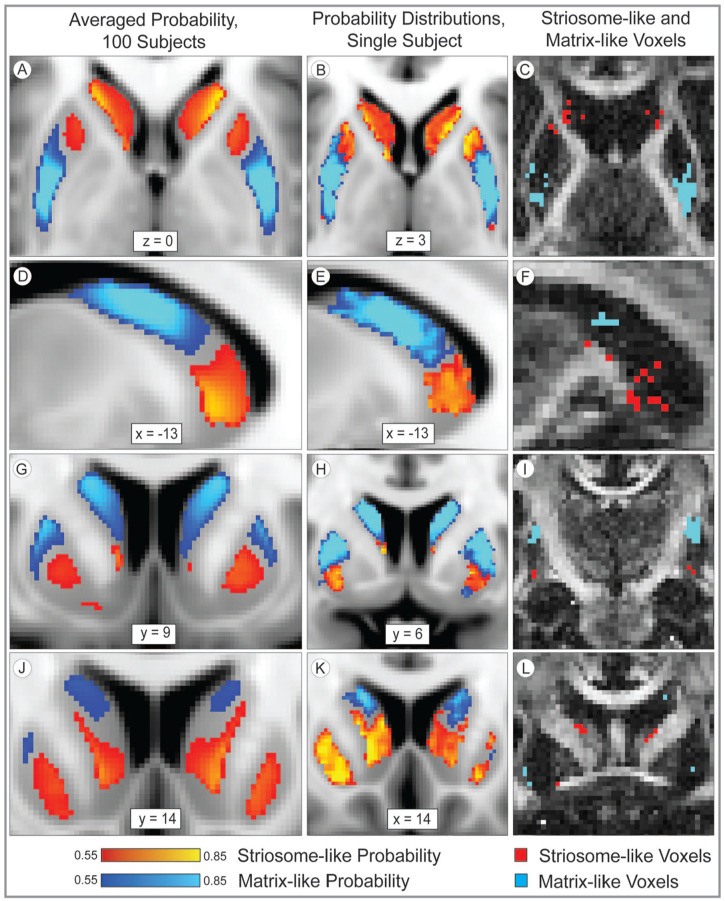
The distribution of striosome-like and matrix-like voxels matches the pattern expected from histology. The averaged probability distributions (left column: A, D, G, J) for striosome-like and matrix-like structural connectivity demonstrate that striosome-like probability (red-yellow) is enriched in the rostral, medial, and ventral striatum, while matrix-like probability (blue-light blue) is enriched in caudal, lateral, and dorsal striatum. This spatial pattern holds true for individuals as well (middle and right columns, all panels from the same subject: B, E, H, K). Striosome-like and matrix-like voxels (right column) are the most-biased voxels from each individual’s striosome-like and matrix-like probability distributions (middle column: C, F, I, L). The middle and right columns illustrate the same planes of section (in standard and native space, respectively) for this individual subject. Note that low-probability voxels (those near the 0.55 probability cut-off) are likely sampling a mix of striosome and matrix tissue. The highly biased voxels of the 1.5SD masks (right column) are substantially more specific and follow the dispersed pattern expected for striosome-like voxels. Probabilities are illustrated relative to the MNI_152_1 mm standard in the left and middle columns. Compartment-like voxels (right column) are illustrated in native space (1.5 mm isotropic resolution) relative to that individual’s fractional anisotropy image. Coordinates follow MNI convention. Images follow radiographic convention.

The typical location of striosome and matrix within the mammalian striatum is remarkably similar across species. In human^30,48,49^ and animal tissue,^27,29,30,50,51^ each compartment is arrayed in medio-lateral, rostro-caudal and dorsal-ventral gradients. Thus, while the striosomal labyrinth is present throughout the striatum, its branches are enriched in medial, rostral, and ventral sites.^52^ This connectivity-based parcellation method identified these same gradients: on average, striosome-like voxels are located more medial, more rostral, and more ventral than matrix-like voxels (Figure 1). As in striatal histology, one may find striosome-like or matrix-like voxels at any selected point in the striatum—but each compartment is biased in its most frequent locations. Note that these assessments of voxel location dealt with the voxels in our equal-volume masks (the most-biased voxels, filling the volume 1.5 standard deviations above the mean), not with every striosome-like and matrix-like voxel. Two factor ANOVA demonstrated that striatal compartment, the striatal nucleus of origin (caudate or putamen), and the interaction of these 2 factors all significantly influenced voxel location. In all planes, these compartment-like voxels matched the location biases demonstrated through prior histology assessments.

The mean location of striosome-like voxels was 1.0 mm more medial (*F*_(1, 102)_ = 1,166; *P* = 1.2 × 10^−57^), 4.0 mm more rostral (*F*_(1, 102)_ = 1,629; *P* = 1.6 × 10^−64^), and 6.2 mm more ventral (*F*_(1, 102)_ = 26,512; *P* = 4.5x10^−125^) than the mean location of matrix-like voxels. Origin within the caudate or putamen also had a significant influence on voxel location. Matrix-like voxels in the putamen were more lateral (*F*_(1, 102)_ = 29.3; *P* = 4.1 × 10^−7^), more caudal (*F*_(1, 102)_ = 139; *P* = 9.3 × 10^−21^), and more dorsal (*F*_(1, 102)_ = 20.7; *P* = 1.5 × 10^−5^) than matrix-like voxels in the caudate. Note that the relative positioning of each nucleus within the hemisphere did not drive these differences in location, as individual voxels were assessed relative to the centroid of their nucleus of origin. However, differences in the size and shape of the caudate and putamen may have allowed for more eccentric voxel placement in the putamen.

The interaction of compartment and nucleus of origin had a significant effect on voxel location in the *y*- (*F*_(1, 102)_ = 45.9; *P* = 8.2 × 10^−10^) and *z*-planes (*F*_(1, 102)_ = 285; *P* = 2.7 × 10^−31^). Simple main effects analysis of the compartment-nucleus interaction showed that in the y-plane, nucleus of origin had a significant effect on matrix location (*F*_(1, 66,709)_ = 146; *P* = 1.4 × 10^−33^), while in the *z*-plane, nucleus of origin had a significant effect on striosome location (*F*_(1, 66,709)_ = 286, *P* = 5.0 × 10^−63^).

### The relative abundance of striosome-like and matrix-like voxels

In human tissue, striatal MSNs are present in a ratio of approximately 15% striosome, 85% matrix.^49^ This ratio is relatively constant across all mammalian species reported to date, despite the many-fold increase in both brain size and total striatal volume from rodent to human. Of voxels that were highly biased (*P* ⩾ .87), striosome-like voxels comprised 12.2% and matrix-like voxels comprised 87.8% of striatal volume (SEM, ±0.68%). The volume of the striosome-like and matrix-like compartments was not meaningfully different from the ratio expected from histology.

### Compartment-specific biases in corticostriate structural connectivity

Injected tract tracers in animals demonstrated that axons originating in primary motor cortex project almost exclusively to the matrix.^27,53-55^ Similarly, axons from posterior orbitofrontal cortex are strongly biased toward the striosome.^28,41,56^ Parcellating the human striatum based on differential connectivity replicates these and other patterns of bias originally established in animals, though in different subjects and using a different diffusion imaging protocol.^14^ We performed N-1 (“leave 1 out,” tractography round 2) striatal parcellations, generated 1.5SD masks from these parcellations, and then assessed compartment-selective connectivity bias using the left-out regions as seeds and compartment-like voxels as targets (tractography round 3). Within each cortical seed region, quantification of high probability connectivity (*P* ⩾ .87 to either compartment-like mask) demonstrated that 95.4% of biased primary motor cortex voxels favored matrix-like striatal voxels (*P* = 4.2 × 10^−85^). Similarly, 98.3% of biased voxels in the posterior orbitofrontal cortex favored striosome-like voxels (*P* = 1.1 × 10^−90^). Our striosome-like and matrix-like parcellations matched the structural connectivity profiles demonstrated previously through tract tracing studies in animals.

Parcellating striatal voxels based on differential structural connectivity replicates many anatomic features of the striosome and matrix: striosome-like and matrix-like voxels match the spatial distribution, relative abundance, and region-specific biases in connectivity expected from striatal histology. However, readers should remember that while this method identified voxels that share many properties of the striatal compartments, these indirect and probabilistic parcellations are not the equivalent of directly identifying striosome and matrix tissue.

### Mapping insulo-striate streamlines

**Figure 2.**
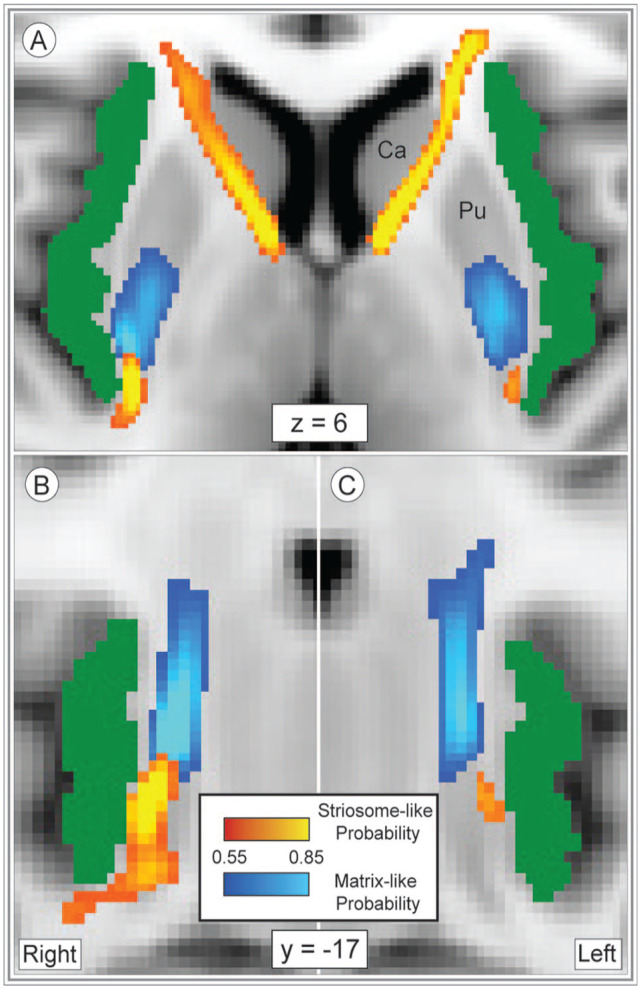
Insulo-striate streamlines followed distinct paths to reach striosome-like vs. matrix-like voxels. Streamlines bound for striosome-like voxels (red-yellow) or matrix-like voxels (blue-light blue) are illustrated in the axial (A) or coronal plane (B), right; C, left; normalized amplitude: 0.5–1.0. Our whole-insula mask is shown in green. Striosome-bound and matrix-bound streamlines followed highly similar paths in the left and right hemispheres, though we generated tractography in each hemisphere independently. Streamlines that originated in the rostral insula that targeted striosome-like voxels exited the insula laterally and traversed the corona radiata and anterior limb of the internal capsule (a). Streamlines that targeted matrix-like voxels, and caudoventral striosome-bound streamlines, exited the insula medially and predominantly transited via the external capsule (A-C). Coordinates follow MNI convention. Images follow radiographic convention. Abbreviations: Ca, Caudate; Pu, Putamen.

We compared the amplitude and location of streamline bundles that linked the insula and either striosome-like or matrix-like striatal voxels (tractography round 4; Figure 2). Total streamlines contacting matrix-like voxels did not differ between left and right hemispheres (right, +10.6%, *P* = 0.11). Streamlines that contacted striosome-like voxels were divided into rostral and caudal bundles, which were assessed separately. Total streamlines in the rostral striosome-favoring bundle were substantially more abundant in the left hemisphere (2.6-fold larger; *P* = 5.9 × 10^−11^). In the caudal striosome-favoring bundle total streamlines did not significantly differ between the hemispheres (right, +33.2%; *P* = .31). Note that the mean insular mask volume differed between the hemispheres by only 48 voxels (1.9% of total insular volume). This small insular asymmetry is not a plausible cause of the large, compartment- and location-specific differences between the hemispheres. Streamlines that contacted striosome-like voxels were 2.7-fold more abundant than those that contacted matrix-like voxels (striosome-like: 1.1 × 10^5^, matrix-like: 4.1 × 10^4^; *P* = 1.2 × 10^−11^).

Next, the locations of compartment-specific insulo-striate streamlines were assessed within the subcortical white matter (Figure 2). Though tractography was performed independently in each hemisphere, the location of streamline bundles was highly similar in the left and right hemispheres (Figure 2A-C). Streamlines bound for matrix-like voxels exited the insula in a single bundle and appeared to transit within the external or extreme capsules (difficult to distinguish at this resolution). In contrast, streamlines bound for striosome-like voxels transited within distinct rostral and caudal bundles (Figure 2(C-E). While the caudal striosome-bound bundle exited the insula medially and transited the extreme/external capsule (paralleling the matrix-bound bundle, Figure 2A-C), the rostral striosome-bound bundle exited the insula from the rostro-lateral surface and transited within the corona radiata and anterior limb of the internal capsule. The rostral striosome-bound bundle appeared to primarily target the caudate, while the posterior striosome-bound bundle appeared to primarily target the putamen.

Insulo-striate streamlines that contacted striosome-like voxels were spatially segregated from streamlines that contacted matrix-like voxels. The core of each streamline bundle was isolated (retaining the uppermost 25%, 50%, or 75% of the streamline bundles, thresholds were previously demonstrated to isolate the core of streamline bundles)^45^ and assessed the overlap of the core striosome-bound and matrix-bound bundles. The uppermost 25% and 50% of striosome-bound and matrix-bound bundles had no voxels overlapping in either hemisphere. Bundles that included the uppermost 75% of voxels had no overlap in the left hemisphere, and only 21 voxels overlapped in the right hemisphere (DSC = 1.73%).

Striosome-favoring and matrix-favoring streamlines were highly segregated when assessed at a voxelwise level, without amplitude thresholds, as well (FSL *randomise*). Following stringent familywise-error and Bonferroni correction, 66.8% of the voxels in our subcortical bounding mask were significantly biased toward 1 of the 2 compartments. These significant clusters closely approximated the mean distributions seen in Figure 2A-C. The striosome-favoring distribution occupied a larger fraction of subcortical voxels (47.1%) than the matrix-favoring distribution (19.7%). The streamline density within these significant clusters underscored the marked difference in subcortical location for compartment-specific streamlines: within the striosome-favoring significant clusters (60 432 voxels), 9161 streamlines/voxel contacted striosome-like voxels, while 305 streamlines/voxel contacted matrix-like voxels; within the matrix-favoring significant cluster (25 319 voxels), 318 streamlines/voxel contacted striosome-like voxels, while 3831 streamlines/voxel contacted matrix-like voxels.

### Spatial distribution of compartment-specific connectivity biases within the insula

**Figure 3.**
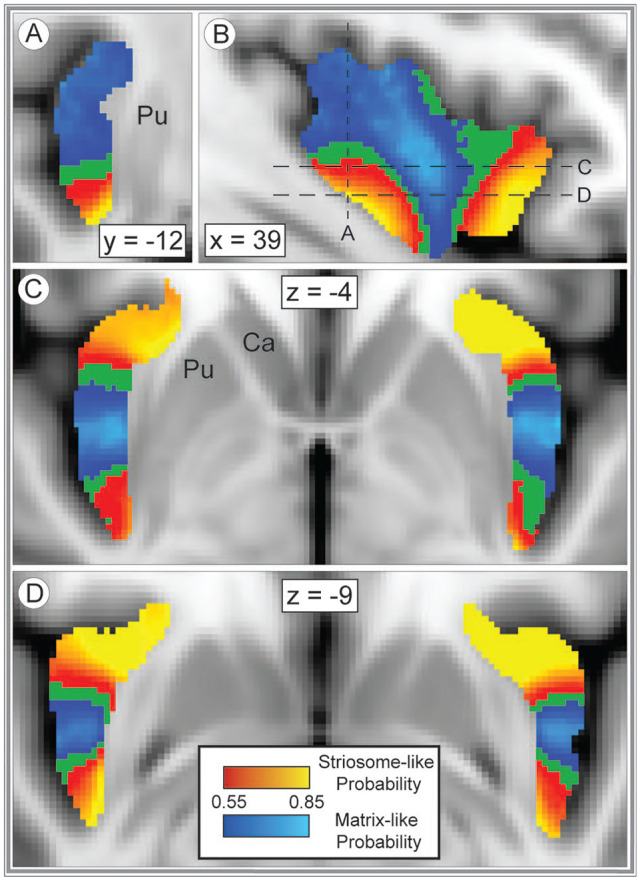
Insulo-striate connectivity with the striosome- or matrix-like compartments is highly segregated. Insular voxels whose connectivity favored striosome-like voxels (red-yellow) or matrix-like voxels (blue-light blue) are overlaid on the insular seed mask (green). All voxels were visualized with probability thresholds of .55 to −.85, revealing the insular mask in minimally-biased voxels (those with *P* < .55). The highest connection probabilities are shown in yellow and light blue for striosome and matrix-like connectivity, respectively. Coronal (A, right hemisphere), sagittal (B, right hemisphere), and axial (C, D) views reveal the distinct zones of insulo-striate structural connectivity. The planes of visualization in A, C, and D are represented by dashed lines in B. Coordinates follow MNI convention. Images follow radiographic convention. Abbreviations: Ca, Caudate; Pu, Putamen.

To map connectivity for each insular subregion, insulo-striate connectivity was assessed at each insular voxel using classification targets mode (tractography round 5). Insular voxels that seeded streamlines bound for striosome-like voxels were largely segregated from insular voxels whose streamlines reached matrix-like voxels (Figure 3). The root-mean-square (RMS) distance between the centers of gravity (COG) for striosome-favoring and matrix-favoring probability distributions was calculated for each individual and hemisphere. Given the visible separation of rostral and caudal striosome-favoring probability clusters (Figure 3), the analyses compared matrix COG with the rostral striosome COG, and matrix COG with the caudal striosome COG. The matrix-favoring cluster was significantly closer to the centroid of the insula than both striosome-favoring clusters (rostral striosome- favoring cluster: 11.0 mm greater RMS distance, *P* = 1.9 × 10^−118^; caudal striosome-favoring cluster: 8.5 mm greater RMS distance, *P* = 6.1 × 10^−111^). Relative to the matrix-favoring cluster, the rostral striosome-favoring probability cluster was 16.8 mm more rostral, 2.0 mm more medial, and 7.7 mm more ventral within the insula (*P*-values, respectively: 1.1 × 10^−109^, 1.3 × 10^−36^, 2.2 × 10^−67^). Relative to the matrix-favoring cluster, the caudal striosome-favoring probability cluster was 11.7 mm more caudal, 2.4 mm more lateral, and 7.0 mm more ventral within the insula (*P*-values, respectively: 2.4 × 10^−84^, 6.4 × 10^−82^, 2.1 × 10^−60^).

### Compartment-specific biases among the insular subregions

**Figure 4.**
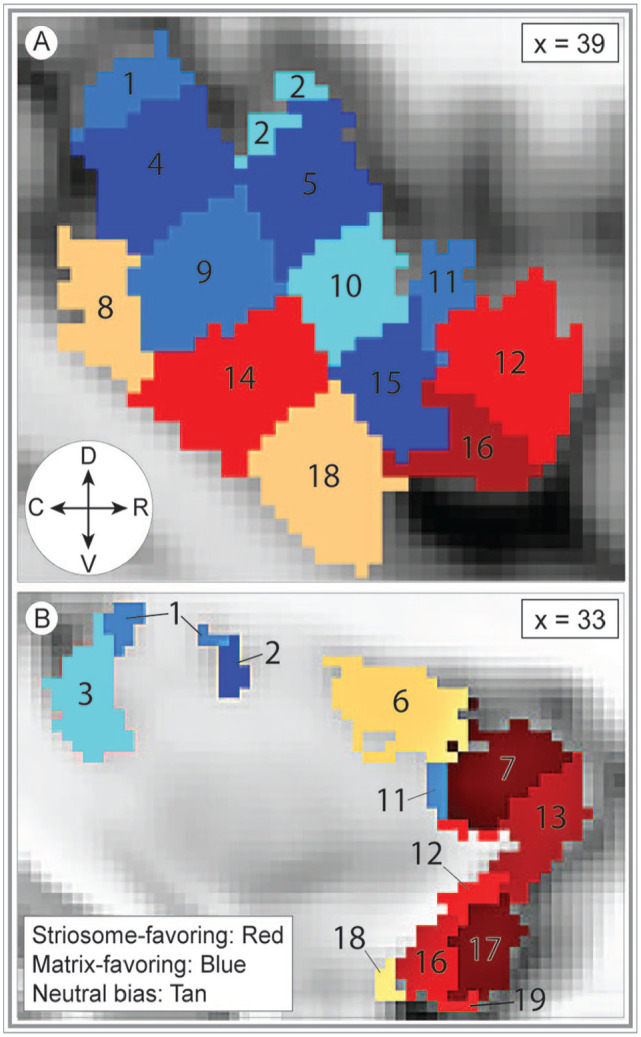
Bias in striatal connectivity clusters among neighboring insular subregions. The 19 masks utilized to extract subregion-specific connectivity estimates are superimposed on the MNI152_T1_1 mm template brain. The ROIs shaded red represent striosome-biased insular subregions and are generally more rostral and ventral. The ROIs shaded blue represent matrix-biased subregions and are generally more caudal and dorsal. The variation in color of the ROIs within each red or blue region is only to distinguish ROI subregions—shading does not indicate the magnitude of bias toward either compartment. The ROIs shaded tan represent subregions with no compartment-specific bias. ROI numbers correspond to those in the bar graph, Figure 5. The sagittal planes of visualization (*x* = 39 or 33) are lateral (A) and medial (B) views, respectively, of the left insula. The compass corresponds to the anatomical direction within the figure (D: dorsal, V: ventral, R: rostral, C: caudal). Coordinates follow MNI convention.

Next, compartment-specific connectivity was quantified (tractography round 5) for each insular subregion in each hemisphere (Figure 4, Supplemental Video 1). For every subregion, compartment-specific connectivity biases matched in the left and right hemispheres. Therefore, both hemispheres were included in subsequent assessments of connectivity bias. Compartment-specific connectivity was quantified in 2 ways. First, for each subregion we extracted the volume with biased connectivity (the number of voxels with probability >.55 for striosome- or matrix-favoring connectivity) and assessed the influence of striatal compartment on connectivity using ANCOVA, with a range of demographic and anatomic factors as covariates (detailed in Section 2.11). This approach investigated the influences on compartment-specific structural connectivity within biased voxels. Second, voxelwise, non-parametric comparisons (striosome-favoring vs. matrix favoring probability distributions, using *randomise*) were performed to identify voxels with significant biases in structural connectivity (familywise error corrected for multiple comparisons). Within each insular subregion, the number of significantly biased voxels was extracted as a fraction of all voxels in that subregion. Comparing the results of these 2 approaches provided anatomic detail not evident in single measures of mean connectivity across a subregion.

**Figure 5.**
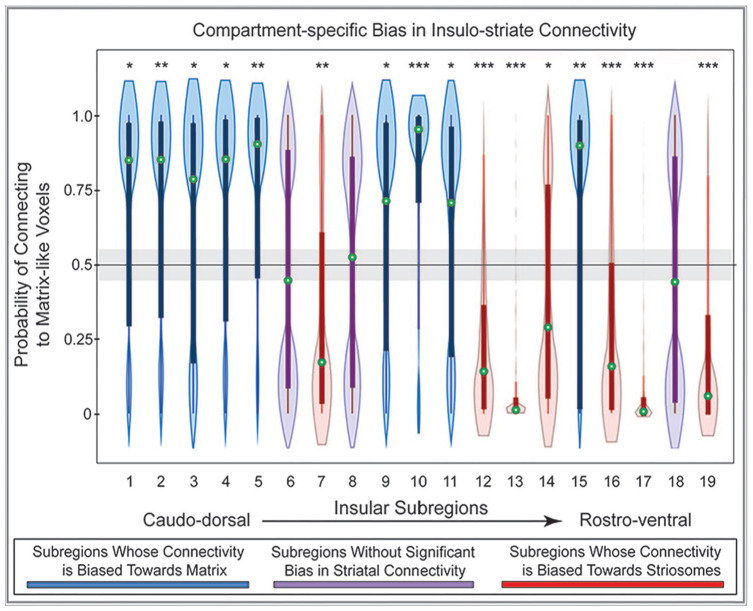
Structural connectivity between 19 insular subregions and the striatal compartments is largely biased toward either striosome-like or matrix-like voxels. Insular subregions are presented from the caudo-dorsal to the rostro-ventral insula (left-to-right). Values higher than 0.55 on the *y*-axis (above the gray bar) indicate matrix-like connectivity bias; values lower than 0.45 (below the gray bar) indicate striosome-like connectivity bias. Nine insular subregions were significantly biased toward matrix-like voxels (blue violin plots), 7 subregions were significantly biased toward striosome-like voxels (red violin plots), and 3 subregions had no significant compartment bias (purple violin plots). Green circles indicate the median for that subregion. Box and whisker plots are superimposed on each violin plot. The values for matrix-like and striosome-like connectivity for any individual insular subregion always sum to one. Therefore, only values for matrix-like connectivity for each insular subregion are presented. Significance thresholds: *P = 2.6 × 10^−3^; **P = 2.6 × 10^−9^; ***P = 2.6 × 10^−15^.

The majority of insular subregions (16 of 19; 84%) were significantly biased toward either striosome-like or matrix-like voxels (Figure 5, Table 2). In every case, the biases identified through ANCOVA and voxelwise testing concurred. Nine subregions were significantly biased toward matrix- like compartments: subregions 1, 2, 3, 4, 5, 9, 10, 11, 15. Seven subregions were significantly biased toward striosome-like voxels: subregions 7, 12, 13, 14, 16, 17, 19. Three subregions had no significant bias in structural connectivity: subregions 6, 8, and 18. Insular subregions with bias toward striosome- like voxels tended to be in rostral and ventral subregions, while those with bias toward matrix-like voxels tended to be in caudal and dorsal subregions (Figure 4). Subregions without significant bias straddled the boundaries between striosome- and matrix-favoring insular zones, and thus included large numbers of voxels that were biased toward either compartment (Figure 5, purple violin plots).

**Table 2.** Compartment-Specific Biases in the Insular Subregions.

Insular subregion	Compartment bias	Results of ANCOVA testing			Results of *randomise* testing	
		% of biased voxels favoring matrix	*F* statistic (*F*_1,379_)	*P*-value	% of subregion voxels with matrix bias	% of subregion voxels with striosome bias
Subregion 1	Matrix-like	65.9	71.2	*P* = 6.9 × 10^−16^	63.6	0.0
Subregion 2	Matrix-like	66.9	71.2	*P* = 4.1 × 10^−18^	85.4	0.0
Subregion 3	Matrix-like	61.4	33.0	*P* = 1.9 × 10^−8^	32.5	0.0
Subregion 4	Matrix-like	65.0	61.7	*P* = 4.3 × 10^−14^	70.1	0.0
Subregion 5	Matrix-like	71.0	144.3	*P* = 2.2 × 10^−28^	82.7	0.0
Subregion 7	Striosome-like	33.6	91.4	*P* = 1.5 × 10^−19^	0.0	63.4
Subregion 9	Matrix-like	60.0	26.8	*P* = 3.7 × 10^−7^	50.7	2.9
Subregion 10	Matrix-like	76.7	231	*P* = 5.0 × 10^−41^	93.6	0.0
Subregion 11	Matrix-like	58.8	22.6	*P* = 2.8 × 10^−6^	51.0	0.18
Subregion 12	Striosome-like	21.9	542	*P* = 4.9 × 10^−75^	1.3	74.6
Subregion 13	Striosome-like	9.3	1,723	*P* = 4.9 × 10^−143^	0.0	98.4
Subregion 14	Striosome-like	40.4	27.8	*P* = 2.2 × 10^−7^	16.3	43.3
Subregion 15	Matrix-like	68.4	88.9	*P* = 4.2 × 10^−19^	79.4	1.9
Subregion 16	Striosome-like	28.9	178	*P* = 1.7 × 10^−33^	5.6	78.0
Subregion 17	Striosome-like	7.3	2,797	*P* = 5.0 × 10^−177^	0.0	97.2
Subregion 19	Striosome-like	21.1	403	*P* = 1.4 × 10^−61^	0.0	82.8

Each insular subregion is numbered to match the insular subregion masks defined by Ghaziri et al^35^ (illustrated in Figure 4). “Compartment Bias” indicates the compartment that is the primary target of streamlines seeded from within that subregion. ANCOVA testing (middle 3 columns) assessed compartment-biased voxels for factors that influenced that bias. For these tests, percentages from 55% to 100% indicate a progressively increasing bias toward matrix-like voxels, and percentages from 45% to 0% indicate a bias toward striosome-like voxels. Voxelwise testing (*randomise*, right 2 columns) assessed all insular voxels for significant bias (familywise error-corrected significance threshold, *P* = .025), expressed as the percent of a subregion’s total volume that was significantly biased toward either compartment. While some subregions included significant voxels for both compartments (eg, subregion 14), 10 of 19 subregions included only voxels biased toward 1 compartment. Note that subregions 6, 8, and 18 were not significantly biased and thus are not included here.

Supplemental Video 1: A video illustrating the left insula, our whole-insula seed mask, and the insular subregion masks that were used to map compartment-specific bias in insulo-striate connectivity. Subregion masks were generated by Ghaziri et al.^35^

Note that comparing the results of ANCOVA and voxelwise testing is useful to distinguish between punctate areas of bias and broader patterns of bias that involve most or all of a subregion. For example, ANCOVA testing (the percent of biased voxels) yielded similar results for subregions 2 and 3, with roughly 2:1 biased voxels (matrix:striosome). Assessing the results of voxelwise testing, however, demonstrated that 85.4% of all voxels in subregion 2 were biased toward matrix-like targets, while in subregion 3 only 32.5% of voxels were significantly biased toward matrix-like targets—and 67.5% of subregion 3 voxels were not significantly biased toward either compartment. Therefore, while streamlines seeded in subregions 2 and 3 were both significantly biased toward matrix-like voxels, this pattern is much more broadly-based in subregion 2.

Demographic and experimental variables were included in our ANCOVA testing to determine the impact of these factors on compartment-specific bias in insulo-striate connectivity. When tested in isolation, age, sex, self-identified race, handedness, and the hemisphere of origin did not have a significant influence on compartment-specific bias, for any region. However, given the recent findings by Cabeen et al^47^ that the histologic structure of the frontal insula is partially determined by hemisphere and subject sex—a secondary analysis was performed for the interaction of sex and hemisphere on compartment-specific bias. Of the 7 rostral subregions that approximated the frontal insula (as mapped by Cabeen et al^47^) the sex-by-hemisphere interaction was a significant contributor to compartment bias in 3 subregions: subregion 11, *F*_(4,379)_ = 8.2, *P* = 2.2 × 10^−6^; subregion 13, *F*_(4,379)_ = 15.4, *P* < 1.2 × 10^−11^; subregion 19, *F*_(4,379)_ = 8.8, *P* = 8.0 × 10^−7^. For 3 additional rostral subregions, the sex-by-hemisphere interaction trended toward significance: subregion 7, *F*_(4,379)_ = 3.7, *P* = 5.3 × 10^−3^; subregion 12, *F*_(4,379)_ = 3.1, *P* = 1.6 × 10^−2^; subregion 17, *F*_(4,379)_ = 3.0, *P* = 1.7 × 10^−2^. In contrast, for the 12 regions that did not approximate the frontal insula, no region reached or trended toward significance.

### The influence of nucleus of origin and rostro-caudal position on insulo-striate connectivity

While compartment-specific biases may explain the spatially segregated patterns of connectivity described here, alternate anatomic factors may influence insulo-striate connectivity as well. We considered the hypothesis that the nucleus of origin—whether a striosome-like or matrix-like voxel resided in the caudate or putamen—influenced the strength or specificity of compartment-specific insular subregion bias. Using the output of tractography round one (striatal parcellation), a second round of striosome-like and matrix-like masks was generated that were proportional to the relative volume of the caudate and putamen. That is, voxels were selected based on the strength of the bias in connectivity, but rather than selecting the 180 voxels with the largest compartment-specific biases (utilized in tractography rounds 1 through 5), caudate and putamen each had a quota of highly biased voxels based on the relative volume of caudate and putamen. Therefore, these striatal masks were biased in their connectivity, but not as biased as those whose sole criteria for selection was connectivity, independent of whether they resided in caudate or putamen. Insulo-striate CTT was then completed with these proportionate striatal masks as the targets for CTT (tractography round 6). Relative to tractography round 5, no subregion began with bias toward 1 compartment and shifted to bias toward the opposite compartment. When ranked by the amplitude of bias, biased subregions in the proportionate masks condition followed the same order as observed in the original 1.5SD masks—with 1 exception, in which 2 subregions differed by 1% in the original and proportionate masks conditions. With proportionate striatal masks, all insular subregions were slightly more biased toward striosome-like voxels (shifting by 0.92%-9.2%, mean: 5.8% shift). This change to proportionate target masks led subregions 3 and 9 to fall from slight matrix-bias (Figure 5) to neutral. No subregion shifted to greater matrix-biased connectivity. One insular subregion was significantly more biased toward striosome-like voxels with proportionate masks (subregion 19, shifting from 21.1% matrix bias to 11.9% matrix bias, *P* = 6.2 × 10^−4^)—all other shifts in bias toward striosome-like voxels were non-significant. We conclude that the magnitude of compartment-specific bias was a more important driver of insulo-striate connectivity than whether a compartment-like voxel was found in caudate or putamen.

The insulo-striate biases identified here share a spatial organization with the striatum: both striosome-like voxels and striosome-favoring insulo-striate projection are enriched in the rostral and ventral portions of their region, with matrix-favoring and matrix-like voxels enriched centrally. Therefore, the possibility was considered that these biases reflect simple spatial ordering of streamlines, an effect of the “neighborhood” in which a voxel resides rather than its compartment-specific bias. This possibility was addressed by jittering the location of the voxels of our striosome-like and matrix-like masks and then comparing our initial tractography (which targeted our precisely-selected compartment-like masks) with tractography that targeted these location-shifted voxels. Notably, the location of each voxel was shifted individually, and by a small, random distance (±0-3 voxels) in each plane. The mean location shift for individual voxels was 1.9 voxels in each plane. However, as the direction of shift was random for each voxel and in each plane, the mean location shift was negligible (<1 mm difference for each plane—*x*: 0.16 mm shift, *y*: 0.10 mm shift, *z*: 0.79 mm shift). The RMS distance from the centroid of either caudate or putamen was 7.6 mm for our starting compartment-like masks (6.6 mm for matrix, 8.7 mm for striosome), and 7.4 mm for the randomly shifted voxels (7.3 mm for matrix, 7.5 mm for striosome). The proximity of these location-shifted voxels allowed us to distinguish compartment-specific and “neighborhood” influences on insulo-striate connectivity.

Our striosome-like and matrix-like masks had mean connectivity biases of 0.84 and 0.97, respectively (neutral: *P* < .55; complete bias: *P* = 1.0). In contrast, our location-shifted voxels had compartment biases of 0.56 for striosome-adjacent and 0.84 for matrix-adjacent masks. The fact that mean bias changed substantially more in striosome-adjacent than in matrix-adjacent voxels matches expectations based on striatal tissue—since 85% of striatal volume lies within the matrix,^57^ shifting location at random is substantially more likely to locate a voxel occupied by matrix. Shifting the location of a highly-biased matrix-like voxel will likely select a less-biased, but still matrix-like, voxel. In contrast, shifting the location of striosome-like voxels may select a less-biased striosome-like voxel (since their location is relatively clustered) or an abundant matrix-like voxel. Our striosome-adjacent voxels reflected this near-neutral pattern of bias. When tractography targeted these location-shifted voxels (tractography round 7), connectivity was markedly and significantly shifted toward matrix-adjacent masks for all 19 insular subregions (range of increase in bias across 19 subregions: 0.60-0.98, 0.21-0.56 greater than prior matrix-like bias; for comparisons of precise-to-shifted targets, *P*-values ranged from 4.7 × 10^−16^ to 2.7 × 10^−56^). Though individual compartment-adjacent voxels were shifted <2 voxels from their precisely-selected starting points, and the mean location of these location shifts was <1 mm, the striosome-favoring insular subregions lost all bias toward striosome-adjacent voxels. The biases identified in insulo-striate projections were driven by the compartment-specific differences in structural connectivity, not by their relative “neighborhood” within the striatum.

## Discussion

### Overview of results

Structural connectivity was assessed in 100 healthy human subjects to determine whether and where insulo-striate projections are biased toward 1 of the 2 striatal compartments, striosome or matrix. Voxels with striosome-like or matrix-like structural connectivity matched the anatomic properties of striosome and matrix demonstrated through histology: their typical locations within the striatum, relative abundance, somatotopy, and the compartment-specificity of cortico-striate projections. Insulo-striate streamline bundles followed segregated paths to reach the striatum, depending on if they targeted striosome-like or matrix-like voxels. The results indicate that insular subregions whose streamlines primarily contacted striosome-like voxels were spatially segregated from subregions whose streamlines primarily contacted matrix-like voxels: 16 of 19 insular subregions had a significant bias toward 1 striatal compartment. Though tractography was generated independently in the left and right hemispheres, these subregion biases and the locations of compartment-specific streamline bundles were highly similar between hemispheres. Biases in insulo-striate structural connectivity were specific for the precisely selected striatal voxels in our striosome-like and matrix-like masks and these biases were not shared by voxels in their immediate vicinity. Our quantitative and qualitative structural connectivity results were compared with prior histologic and imaging studies in human, primate, and non-primate animal studies. Our diffusion MRI-based results matched the compartment-specific biases in structure and function described in multiple species through diverse experimental methods.

### Limitations of connectivity-based striatal parcellation

While probabilistic tractography is a powerful tool for understanding structural connectivity non-invasively in living organisms, this technique has notable limitations. Tractography cannot distinguish between afferent and efferent connections, cannot detect synapses, and is limited by the millimeter-scale resolution of diffusion MRI signal acquisition. Image resolution is a specific and important limitation of our parcellation method: the size mismatch between striosome branches (0.5-1.25 mm in diameter in coronal sections)^30,49^ and our diffusion voxels (1.5 mm isotropic) assures that each striosome-like voxel includes some fraction of matrix tissue. Therefore, our quantitative assessments were restricted to only the most-biased voxels, those whose connection bias was ⩾1.5 standard deviations above the mean. While this approach replicated many anatomic features of striosome and matrix—their spatial distribution, relative abundance, somatotopy, and their patterns of extra-striate connectivity—readers should bear in mind that our method did not directly identify striosome and matrix tissue. Higher resolution diffusion acquisitions will reduce, but not eliminate, this mixed-sampling limitation.

Imperfections in the segmentation or registration of our regions of interest and bounding masks (discussed in Methods) may have reduced the anatomical precision of our tractography. However, the test-retest accuracy of our histologically-based striatal parcellation method (99.8%)^14^ suggests that such imperfections have a small influence on parcellations. Another potential source of error in our approach is the coexistence of MSN populations and *en passant* white matter bundles within striatal voxels. Jones and Cercignani^58^ estimated that up to 90% of brain voxels may contain multiple fiber pathways; this is especially problematic in the striatum, with its intercalated descending cortical projections. For this reason, modeling crossing fibers was essential (using the FSL tool *bedpostX*, which estimates tensors for each of 3 principal diffusion directions). Notably, alternate models for estimating diffusion fibers may perform better than the tensor-based tools utilized in this study, including models that utilize fiber orientation distribution functions^59^ or Q-ball imaging,^60^ available through the widely-adopted freeware tools MRtrix and DSI Studio, respectively. While these limitations to probabilistic tractography must be considered, at every point of comparison that these tractography-based results were consistent with prior histologic and MRI-based assessments of the insula and the striatal compartments. Finally, the insular segmentations utilized in this study are not the only reasonable method for subdividing this structure. Researchers interested in alternative insular subregions could extract measures of connectivity bias from our existing insular probability maps, tailoring these methods to their own investigations.

### Network implications of segregated insulo-striate connectivity

There is substantial evidence that cortical regions and sub-portions of subcortical nuclei are involved in distinct functional and structural networks. For example, patients with lesions in the pulvinar nucleus of the thalamus exhibited chronic neuropathic pain, whereas patients with lesions concentrated in adjacent thalamic nuclei had no pain symptoms.^61^ Electrical stimulation of the dorso-caudal insula produced somatic pain, with insular somatotopy corresponding to specific body sites.^62^ Insular functional activation increases following heat-induced^2^ and capsaicin-induced pain^63^ specifically in this same dorso-caudal insular site. In both the anterior pulvinar^40^ and dorso-caudal insula, structural connectivity is significantly biased toward matrix-like voxels. Whether the dorso-caudal insula and anterior pulvinar nucleus act in concert to mediate pain in humans, with the matrix as an obligate network hub, will require future investigation. Insulo-thalamic connectivity has been shown to operate within distinct CSTC loops.^64^ The anterior insula operates in a discrete regulatory circuit with the dorsal striatum and mediodorsal thalamus across a broad range of psychiatric disorders.^65^ Similarly, reward cues activate the ventral striatum, rostral insula, and caudal thalamus (as a group), but non-reward cues do not.^66^ The rostral orbitofrontal cortex shares a distinct functional connectivity network with the insula and mediodorsal thalamus.^67^ The functional connectivity between these insular subregions and diverse brain areas supports the premise that insular subregions may operate within distinct CSTC loops. The highly segregated routes followed by striosome-bound and matrix-bound insulo-striate streamline bundles (Figure 2) underscore the premise that insular subregions may be embedded within distinct CSTC loops, mediated in part through segregation by striatal compartment.

Prior studies that injected anterograde tracers in the agranular and frontoinsular cortex of the cat, macaque, or mouse demonstrated preferential projections to the striosome, with minor projections to the matrix.^28,32,68^ Our results in humans concur with these histologic studies: streamlines seeded in the rostral and ventral portions of the insula predominantly reach striosome-like voxels. Although direct compartment-specific connectivity of the dorsal and caudal portions of the insula has not been mapped using injected tracers in animals, to the best of our knowledge, cortical regions that were previously shown to be matrix-favoring also selectively projected to the caudal insula.^6-8^ The present results, based on bait regions that were validated through animal tract tracing studies and tested in a large, broadly representative neuroimaging cohort, confirm and extend these prior findings, and suggest that compartment-specific biases in insulo-striate connectivity cluster by insular subregions (Figures 2 and 3).

### Compartment-specific connectivity and insular cytoarchitecture are spatially similar

**Figure 6.**
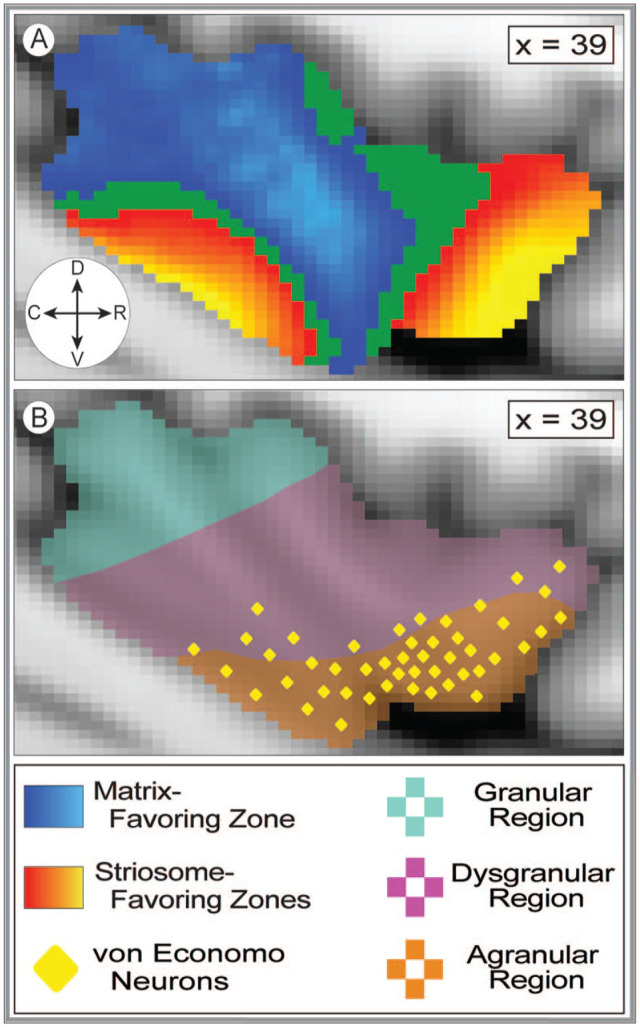
Structural connectivity with striosome-like and matrix-like voxels parallels the cytoarchitectural organization of the insula. (A) represents the regions in the insula with striosome-like (red-yellow) and matrix-like (blue-light blue) connectivity. The yellow and light blue colors correspond to higher degrees of connectivity (with striosome-like or matrix-like voxels, respectively). The insular mask can be seen in the background at points where connectivity was not significantly biased toward one compartment (green voxels). (B) represents the general cytoarchitecture of the human insula based on prior figures.^5,72,80,81^ The rostroventral red region represents the agranular region of the insula, the purple region represents the dysgranular region of the insula, and the blue region represents the granular region of the insula. The yellow diamonds represent the distribution of von Economo neurons in the human insula based on prior figures.^5,72,82^ The plane of visualization for both A and B is sagittal in the right hemisphere at *x* = 39 (MNI coordinates). The compass corresponds to the anatomical direction within the figure (D: dorsal, V: ventral, R: rostral, C: caudal).

Compartment-specific projections may be related to underlying differences in the cytoarchitecture of the rostral vs. caudal, and ventral vs. dorsal insula (Figure 6). Cell density and granularity increases from the rostroventral to caudodorsal insula, generally in a wave-like pattern (Figure 5).^5,69,70^ This cytoarchitectural segregation of the insula aligns with the compartment-specific biases in structural connectivity observed in this study. The rostral agranular area, as well as the central dysgranular area that extends along the ventral rostro-caudal axis of the insula, are spatially colocalized with our rostral and caudo-ventral zones that preferentially project to striosome-like targets.^5,71^ The similarity in cytoarchitecture (agranularity) between the rostral insula and the ventral portion of the dorsal insula (Figure 6) overlaps the insular zones that were significantly biased toward striosome-like voxels (Figure 4). Intriguingly, a second insular gradient, the abundance of von Economo neurons (VENs), also parallels the biases in insulo-striate connectivity observed here (Figure 5).^72,73^ VENs are highly enriched in humans and hominid primates, relative to other mammals. VENs are restricted to a few cortical sites, including the rostral insula.^74,75^ Though the functions of VENs are incompletely understood, they are proposed to play an outsized role in social-emotional cognition.^74-76^ Insular VENs are most abundant in rostroventral subregions and dwindle as one moves caudally and dorsally, similar to the striosome-favoring bias in structural connectivity seen here. No prior studies mapped VEN projections with the striatal compartments, to the best of our knowledge. However, a recent report found that *Lypd1*, a peptide marker of VENs,^77^ is also selectively enriched in the striosome.^78^ If future studies demonstrate that VENs selectively project to the striosome, this association may serve as a substrate for limbic symptoms in compartment-selective neurodegenerative diseases.^19,79^

### Relating biases in structural connectivity to functional specialization

The gradient of granularity from the rostroventral to caudodorsal insula has many similarities with subregional patterns in functional connectivity. The rostroventral insula is part of a functional network that includes the frontal and temporal opercula, inferior frontal gyrus, temporal sulcus, and the anterior cingulate cortex.^83^ Recognized functions involving these regions include processing of emotions, executive functions, and cognitive control tasks.^84-86^ The caudodorsal insula is part of a functional network that includes the primary and secondary somatomotor cortices, parietal opercula, the supplementary motor area, lateral temporal cortex, and the inferior frontal gyrus.^83^ In concert with these regions, the caudodorsal insula mediates motor tasks and somatosensory processing.^3,87,88^

This rostroventral-to-caudodorsal shift in insular functional connectivity closely approximates the shift in compartment-specific biases identified here. Joel et al^89^ argued that the matrix compartment is preferentially responsible for action, whereas the striosome compartment is preferentially responsible for critique of that action. The determination that the rostroventral insula (biased toward striosome-like voxels, Figure 3) is responsible for processing emotions and regulating behavior^4,90^ is consistent with the compartment-specific bias observed here, as the striosome receives selective projections from limbic areas, including the basolateral amygdala,^32^ rostral anterior cingulate,^28^ and prelimbic and infralimbic cortices.^91^ Additionally, prior observations that the caudodorsal insula (our matrix-favoring area) is functionally coupled with areas activated during sensory and motor tasks^83,88,92^ parallels observations in cats, squirrel monkeys, macaques, and mice that primary motor and sensory cortices selectively project to the matrix.^49,51,54,55,93^ The differences in structural connectivity identified here are potential anatomic substrates for the functional specialization of these cytoarchitecturally-distinct zones.

### The influence of hemisphere and sex on compartment-specific projections

Cabeen et al^47^ identified a hemisphere-by-sex interaction in insular development, influencing both cortical surface geometry and microstructure of the frontoinsular cortex (FI).^47^ They observed that women have an increased FI surface area (+7.4%) and volume (+15.3%), relative to men, in the left hemisphere. In parallel, prior fMRI and probabilistic tractography studies identified higher total insulo-striate connectivity in the left hemisphere compared with the right.^8,94^ These findings led us to investigate the interaction of hemisphere and sex on our compartment-specific structural connectivity. Similarly, to these prior reports, striosome-favoring streamlines were significantly increased in the left hemisphere, specific to the rostral insula and driven by subject sex (Figure 2). It is possible that the left hemispheric dominance of insulo-striate connectivity described here may derive from the relative increase in left rostral insula volume described in women.^47^ However, this asymmetry persisted in our streamline measures after correcting for insular volume, suggesting that increased insular volume is not the primary driver of increased structural connectivity in the left insula.

### Insulo-striate dysfunction in disease and injury

This study demonstrated that insular subregions selectively project to somatotopically-defined portions of striosome or matrix. Therefore, injury to either insula or striatum may produce selective functional deficits within insulo-striate networks that manifest as disease-specific symptoms, and injury may be shared among the nodes in this network. In Huntington disease (HD), comorbid striatal and insular atrophy was a potent negative predictor of performance on executive tasks relative to individuals with isolated striatal atrophy.^95^ In patients with Parkinson disease (PD) with mild cognitive impairment, dopamine depletion in the associative striatum was predictive of bilateral dopamine D2 receptor loss in the insula—suggesting that striatal and insular alterations may be interrelated in patients with these symptoms.^96^ Knowledge of compartment-specific insulo-striate connectivity patterns could provide an anatomic substrate to explain the different clinical courses of distinct HD and PD subtypes.

Linked insulo-striate functions may also inform addictive behaviors. When subjects who were avid smokers suffered from strokes that injured the insula, they were significantly more likely to quit compared with smokers with insula-sparing strokes.^97,98^ Intriguingly, the probability of smoking cessation increased when the striatum was damaged along with the insula, compared with injury to non-striatal sites. In individuals suffering from cocaine dependency, functional connectivity between the bilateral putamen and posterior insula/right postcentral gyrus is reduced.^99^ Whether this abnormal insulo-striate functional connectivity is compartment-specific is unknown. However, given that striosome and matrix have opposing responses to striatal dopamine,^24^ and the striosome, but not the matrix, innervates the dopaminergic neurons of the substantia nigra pars compacta,^100^ it is plausible that hyperdopaminergic injury and adaptation are compartment-specific. In rodent models, cocaine-induced dopamine release is significantly different in striosome and matrix, in a pattern that reverses between dorsal and ventral striatum (dorsal: 36% lower in striosome; ventral: 64% higher in striosome).^101^ Methamphetamine exposure leads to a significant depletion of monoamine terminals and neuronal apoptosis,^102^ and methamphetamine-associated injury was 4-fold greater in the striosome than the matrix.^103^ This inter-compartment difference in susceptibility was significantly larger in the medial than the lateral striatum. Since cortico-striate projections to each compartment are somatotopically organized,^28,104^ including in humans,^14^ toxin-associated neural injury may selectively impact particular insulo-striate projections, and thus, the neuropsychiatric functions of particular insular subregions. Further mapping of the somatotopy of insulo-striate projections may localize the insular functions at risk from acquired striatal injury.

### Conclusions

Understanding the networks in which a brain region participates, and the functions those networks subserve, is critical to understanding how that region operates in health and disease. This study demonstrated that closely approximated insular subregions can have markedly different projections to the striatum, based in part on whether streamlines from those subregions target voxels with striosome-like or matrix-like connectivity profiles. These striosome-favoring and matrix-favoring insular zones may therefore be embedded in distinct CSTC loops with opposing responses to striatal dopamine release, differential susceptibility to hypoxic and excitotoxic injury, segregated vascular supplies, and that project to, and receive projections from, different sets of thalamic nuclei. A granular characterization of compartment-specific biases in structural connectivity is an essential step in understanding the functions of the human insula.
